# Polymorphisms in the *SAA*1/2 Gene Are Associated with Carotid Intima Media Thickness in Healthy Han Chinese Subjects: The Cardiovascular Risk Survey

**DOI:** 10.1371/journal.pone.0013997

**Published:** 2010-11-16

**Authors:** Xiang Xie, Yi-Tong Ma, Yi-Ning Yang, Zhen-Yan Fu, Xiao-Mei Li, Ding Huang, Xiang Ma, Bang-Dang Chen, Fen Liu

**Affiliations:** 1 Department of Cardiology, First Affiliated Hospital of Xinjiang Medical University, Urumqi, China; 2 Xinjiang Key Laboratory of Cardiovascular Disease Research, Urumqi, China; VU University Medical Center and Center for Neurogenomics and Cognitive Research, Netherlands

## Abstract

**Background:**

Serum amyloid A protein (SAA) is not only an inflammatory factor, but also an apolipoprotein that can replace apolipoprotein A1 (apoA1) as the major apolipoprotein of high-density lipoprotein (HDL), which has been linked to atherosclerosis. However, the relationship between genetic polymorphisms of SAA and the intima-media thickness (IMT) of the common carotid artery in healthy subjects remains unclear. We investigated the role of *SAA*1 and *SAA*2 gene polymorphisms with IMT in a cohort of healthy subjects participating in the Cardiovascular Risk Survey (CRS) study.

**Methodology/Principal Findings:**

Anthropometric and B-mode ultrasound of the carotid IMT were measured in 1914 subjects (849 men; 1065 women) recruited from seven cities in Xinjiang province, (western China). Four SNPs (rs12218, rs2229338, rs1059559, and rs2468844) were genotyped by use of the polymerase chain reaction-restriction fragment length polymorphism (PCR-RFLP) method. The SNP rs12218 was associated with carotid IMT by analyses of a dominate model (*P*<0.001) and additive model (*P* = 0.003), and the difference remained significant after multivariate adjustment (*P* = 0.008, *P*<0.001, respectively). This relationship was also observed in rs2468844 after multivariate adjustment by recessive model analysis (*P* = 0.011) but this was not observed in rs2229338 and rs1059559 before and after multivariate adjustment. These associations were not modified by serum HDL concentration. Furthermore, there were significant interactions between rs2468844 and rs12218 (interaction *P*<0.001) and rs2229338 (interaction *P* = 0.001) on carotid IMT.

**Conclusion/Significance:**

Both rs12218 of the *SAA*1 gene and rs2468844 of *SAA*2 gene are associated with carotid IMT in healthy Han Chinese subjects.

## Introduction

Inflammation is a key process in the pathogenesis of atherosclerosis (AS). Accumulated evidences suggest that several environmental risk factors for cardiovascular disease (CVD; e.g., smoking, obesity and alcohol) may act by promoting inflammation. Moreover, many epidemiological studies have confirmed that levels of high density lipoproteins (HDL) have a strong inverse relationship with atherosclerosis and coronary artery disease (CAD). In general, a low level of high-density lipoprotein-cholesterol (HDL-C) in plasma is accepted as being a strong and independent risk factor for the development of premature atherosclerosis.

Serum amyloid A (SAA) is not only one of the acute phase proteins, but also a kind of apolipoprotein. SAA is primarily synthesized in the liver by activated monocytes and macrophages [1]. Studies [2] have shown that *in vivo* concentrations of SAA in plasma can be dramatically increased (up to 1000-fold) during acute inflammatory conditions [3]. As an apolipoprotein, SAA is associated with HDL and during inflammation can contribute up to 80% of its apoprotein composition [3]. Many studies have demonstrated that sustained high expression of SAA may contribute to atherogenesis [4], [5], and that an elevated concentration of SAA is associated with an increased risk of CVD [6]. However, the relationships between *SAA* gene polymorphisms and carotid atherosclerosis remain unclear.

The human *SAA* gene cluster on the short arm of chromosome 11, localized to band p15.1, contains four related genes, *SAA*1-4, within a 150-kb region [7]. Only the *SAA*1 and *SAA*2 genes encode acute-phase SAAs (SAA1 and SAA2 proteins), so a lot of research focuses on them. Recently, Carty *et al*. [8] reported an association of *SAA1* and *SAA2* gene polymorphisms and carotid intima-media thickness (cIMT), HDL, and total CVD. However, the relationship between the genetic polymorphisms of *SAA*1/2 and carotid cIMT and HDL level in healthy subjects is incompletely understood.

The aim of the present study was to explore the relationship between *SAA*1/2 gene polymorphisms and cIMT as well as HDL levels in plasma in healthy subjects participating in the Cardiovascular Risk Survey (CRS).

## Results

### Characteristics of study participants

The study cohort consists of 1914 subjects (849 men; 1065 women). The clinical and metabolic characteristics of the study population are shown separately for men and women in Table 1.

**Table 1:** Demographic and risk profile of the study population.

Risk factor	No. (%) or Mean (SD)		
	Total cohort	Men	Women
Never drink (%)	1556 (80.4)	528 (60.9)	1028 (96.0)
Former drinker (%)	82 (4.2)	68 (7.9)	14 (1.3)
Current drinker (%)	276 (14.3)	253 (29.4)	23 (2.1)
Never smoking (%)	1380 (71.3)	319 (37.0)	1058 (98.8)
Former smoking (%)	131 (6.8)	127 (14.7)	4 (0.4)
Current smoking (%)	422 (21.8)	415 (48.1)	7 (0.7)
Age (years)	48.34 (11.99)	49.19 (13.38)	47.65 (10.72)
BMI (kg/m^2^)	24.01 (3.24)	24.62 (2.80)	23.50 (3.48)
SBP(mmHg)	127.5 (17.0)	129.24 (15.59)	126.32 (17.90)
DBP(mmHg)	77.9 (12.0)	79.97 (12.33)	76.42 (11.59)
Uric acid (µmol/L)	298.5 (83.8)	346.36 (81.16)	260.32 (63.75)
Glucose (mmol/L)	5.03 (1.17)	5.17 (1.33)	4.93 (1.01)
Triglyceride (mmol/L)	1.46 (1.17)	1.73 (1.40)	1.25 (0.89)
TC (mmol/L)	4.54 (0.92)	4.55 (0.93)	4.54 (0.92)
HDL –C (mmol/L)	1.40 (0.40)	1.28 (0.38)	1.50 (0.39)
LDL-C (mmol/L)	2.88 (0.88)	2.93 (0.90)	2.83 (0.85)

Note: BMI, body mass index; SBP, systolic blood pressure; DBP, diastolic blood pressure; HDL, high-density lipoprotein; LDL, high-density lipoprotein; TC, total cholesterol.

### *SAA1/2* genotype and allele frequencies

All genotyped SNPs were in Hardy-Weinberg equilibrium and common with minor allele frequencies >0.05. Table 2 shows detailed information for each SNP as well as the allele frequencies.

**Table 2:** Genotypes and alleles distribution of study subjects.

SNPs	n	Genotype (%)			Allele (frequency)	
rs12218	1914	TT	CT	CC	T	C
		1050 (54.9)	734 (38.3)	130 (6.8)	0.74	0.26
rs2229338	1912	AA	AG	GG	A	G
		1583(82.8)	313 (16.4)	16 (0.8)	0.91	0.09
rs1059559	1910	TT	CT	CC	T	C
		1348 (70.6)	513 (26.9)	49 (2.5)	0.84	0.16
rs2468844	1914	AA	AG	GG	A	G
		1520 (83.4)	369 (15.8)	25 (0.08)	0.89	0.11

### Associations with baseline IMT and HDL level

Using general linear model analysis, rs12218 was found to be significantly associated with serum HDL levels in a dominate model or additive model before (*P*<0.001, *P*<0.001, respectively) and after multivariate adjustment (*P* = 0.003, *P* = 0.011, respectively). Also, rs2468844 was significantly associated with serum HDL levels in a dominate model or additive model before (*P*<0.001, *P*<0.001, respectively) and after multivariate adjustment (*P* = 0.003, *P* = 0.011, respectively, Table 3).

**Table 3:** Association of *SAA1* and *SAA2* SNPs with HDL.

		Mean IMT (cm) ± SD			Model 1‡			Model 2§		
SNP	Wild/Rare Allele	Homozygous for Rare Allele	Heterozygous	Homozygous for Wild Allele	*P* (Rec*)	*P* (Dom†)	*P* (Add⋇)	*P* (Rec*)	*P* (Dom†)	*P*(Add⋇)
rs12218	T/C	1.25±0.32	1.29±0.31	1.45±0.45	0.076	<0.001	<0.001	0.065	0.006	0.011
rs2229338	A/G	1.39±0.36	1.41±0.38	1.41±0.39	0.132	0.669	0.678	0.213	0.373	0.179
rs1059559	T/C	1.44±0.27	1.41±0.30	1.41±0.41	0.179	0.306	0.364	0.387	0.128	0.689
rs2468844	A/G	1.28±0.35	1.33±0.32	1.44±0.40	0.036	<0.001	<0.001	0.067	<0.001	0.004

§ analysis of covariance adjusted for sex and age;

‡ Unadjusted model;

*recessive model;

† dominant model;

⋇ additive model.

As shown in Table 4, rs12218 was associated with cIMT in a dominate model or additive model before (*P*<0.001, *P* = 0.003, respectively) and after multivariate adjustment (*P* = 0.008, *P*<0.001, respectively). However, rs2468844 was associated with cIMT only in a recessive model after multivariate adjustment (*P* = 0.011). These associations were not found in rs2229338 and rs1059559 before and after adjustment of key co-variants.

**Table 4:** Association of *SAA1* and *SAA2* SNPs with IMT.

		Mean IMT (cm) ± SD			Model 1‡			Model 2§		
SNP	Wild/Rare Allele	Homozygous for Rare Allele	Heterozygous	Homozygous for Wild Allele	*P* (Rec*)	*P* (Dom†)	*P* (Add^⋇^)	*P* (Rec*)	*P* (Dom†)	*P*(Add^⋇^)
rs12218	T/C	0.086±0.04	0.080±0.03	0.070±0.03	0.068	<0.001	0.003	0.077	0.008	<0.001
rs2229338	A/G	0.082±0.04	0.080±0.03	0.079±0.02	0.754	0.658	0.465	0.549	0.473	0.276
rs1059559	T/C	0.081±0.03	0.079±0.02	0.078±0.02	0.259	0.147	0.324	0.437	0.256	0.423
rs2468844	A/G	0.083±0.03	0.081±0.03	0.081±0.02	0.042	0.060	0.073	0.011	0.038	0.023

Significant interactions were observed between rs2468844 and rs12218 (interaction *P*<0.001) and rs2229338 (interaction *P* = 0.001) on carotid IMT (Table 5).

**Table 5:** Interaction of *SAA1* and *SAA2* on IMT.

Source	Type III Sum of Squares	df	Mean Square	F	P
Corrected Model	0.140^a^	26	0.005	39.271	<0.001
rs12218	0.007	2	0.003	25.040	<0.001
rs2229338	6.005×10^−6^	2	3.003×10^−6^	0.022	0.978
rs1059559	1.859×10^−4^	2	9.294×10^−5^	0.679	0.507
rs2468844	0.001	2	0.001	5.001	0.007
rs12218 * rs2468844	0.012	4	0.003	22.125	<0.001
rs2229338 * rs2468844	0.002	2	0.001	7.342	0.001
rs1059559* rs2468844	4.404×10^−4^	4	1.101×10^−4^	0.804	0.523

a R Squared = 0.352 (Adjusted R Squared = 0.343).

## Discussion

We found that variation in the *SAA*1 and *SAA*2 genes is associated with the IMT of the common carotid artery in healthy Han Chinese subjects. These associations were not modified by the HDL concentration in serum. Furthermore, there were significant interactions between *SAA*1 and *SAA*2 on cIMT. This was the first study investigating common allelic variants in *SAA1* and *SAA2* genes and their association with IMT in Han Chinese subjects.

The foundation for human studies examining putative causative genes that may be involved in cIMT is based on a candidate gene approach. This involves selecting a functionally relevant gene to study and subsequently investigating its association with carotid atherosclerosis. The genes for *SAA*1and *SAA*2 are candidates for atherosclerosis because they are the genes encoding one important inflammation factor, SAA, which is synthesized by the liver [9].

### Plasma SAA levels, serum HDL concentration and carotid IMT

In the early 1970s, SAA was identified as the plasma protein responsible for forming tissue deposits called “amyloid (AA-type)” seen in diseases with underlying persistent acute inflammation [10], [11]. Soon after its discovery, SAA was shown to be an acute-phase protein produced by the liver within hours of tissue injury regardless of cause. Its plasma concentration can increase a 1000-fold within 24 h [12], [13]. In plasma, SAA is associated with HDL [14], [15] and, during severe inflammation, can contribute ≤80% of its apo-protein composition [16], [17]. The displaced apoA-I is rapidly cleared by the liver and kidneys [18], together with a sharp decline in apoA-I gene expression during inflammation [19].

O'Brien et al. [20] demonstrated that SAA accumulates in atherosclerotic lesions of apoE−/− and LDLR−/− mice and suggested that SAA might play a part at each stage of lesion development. Johnson et al. [6] reported a strong independent relationship between SAA and future cardiovascular events. They indicated that systemic inflammation, as manifested by high SAA levels, may promote destabilization of atherosclerotic plaque, in addition to exerting a possible direct effect on atherogenesis. IMT can be used as an independent surrogate marker of atherosclerosis [21]. Therefore, elevated levels of SAA and decreased concentrations of HDL are positively associated with IMT (just as with atherosclerosis).

### SAA1/2 genotypes, plasma SAA levels and serum HDL concentrations

Increasing evidence suggests that inflammation is important in the pathogenesis of atherosclerosis, stroke, and ischemic heart disease. In prospective studies, elevated basal levels of SAA were shown to be predictive of future vascular events [6]. In 2000, Yama et al. [22] reported that the *SAA*1 allele influences the plasma concentration of SAA. In the Japanese population, subjects with the SAA1.5 allele have a higher plasma concentration of SAA than those lacking this allele. In the present study, we did not examine the SAA levels between each genotype, which was a limitation of our analysis. We can consider that this SNP may be associated with the tagging SNP we selected in the present study, although we could not identify the rs number of the SAA1.5 polymorphism described by Yama et al [22]. In the present study, we also found that the *SAA*1/2 polymorphisms were associated with the serum concentration of HDL. This result was in accordance with the finding reported by Carty et al. [8].

In addition, SAA is secreted into plasma, where it associates primarily with HDL particles but also with very-low-density lipoprotein (VLDL) particles [13]. SAA has been shown to mediate the binding of HDL to differentiated macrophages and endothelial cells [23] and to impair the ability of HDL to promote cholesterol efflux from macrophages [24].

### SAA1/2 genetic polymorphisms and cIMT

Carty et al. [8] reported that *SAA*2 SNPs were associated with increased IMT in African-American subjects, but this association was not observed in European- American subjects. In the present study, we found not only *SAA2* SNP (rs2468844) but also *SAA1* SNP (rs12218) was associated with increased IMT in Han Chinese subjects after adjustment for age, sex, and other factors. These two genes also had significant interactions with cIMT. SAA also has the propensity to affect the concentration and composition of HDL, so we also examined whether the relationship between SAA genetic variants and IMT is modified by HDL concentration. There was a strong association between rs12218 and rs2468844 and circulating levels of HDL, but the serum level of HDL was not an independent predictor of cIMT and did not modify the relationship between SAA genetic variants and IMT. Carty et al. investigated the relationship between SAA gene SNPs and HDL levels and IMT in disease states, particularly in subjects with CVD [8]. The population in the present study was a large healthy cohort, so we could evaluate relationships independent of the potentially confounding secondary effects of the disease. However, the present study was cross-sectional; the CRS study is a multicenter study, and we can investigate a large and well-characterized healthy population.

The mechanisms which may link *SAA*1/2 genetic polymorphisms to carotid atherosclerosis are largely unknown. However, it is accepted that SAA can generate HDL using cellular lipids, a function mediated by the ATP-binding cassette transporter proteins ABCA1 or ABCA7. In addition, in the inflammatory state, HDL composition is altered because its apoA-I and apoA-II components are displaced by SAA in the form of predominately SAA protein. ApoA-I plays an important part in reverse cholesterol transport and therefore may be protective against atherosclerosis, so its displacement by SAA is considered to be potentially pro-atherosclerotic. Carty et al. [8] indicated that variation in SAA1/SAA2 could lead to altered binding affinity of the SAA proteins for HDL and that the association between *SAA*1/2 genetic variants and CVD may be modified by HDL. Although neither the study by Carty et al. nor the present study employed the functional analysis of variation in *SAA*1/2, the present study was in accordance with that of Carty et al. with respect to the relationship between *SAA*1/2 and cIMT. Nonetheless, rs2468844 is a non-synonymous mutation and rs12218 is a synonymous mutation. Whether both of these two polymorphisms affect the stability or binding of the protein is not known. Traditionally, silent SNPs have largely been assumed to not exert a discernible effect on the function or phenotype of genes. However, recent reports have changed this common viewpoint. Kimchi-Sarfaty et al. [25] suggested that silent SNPs may affect the translation rates of proteins, hence influencing the folding and activity of proteins. Therefore, silent SNPs that do not change the coding sequence of the protein may contribute to altered gene function. Whether the silent SNPs in the *SAA* gene can affect its function is unclear, but merits further investigation.

In conclusion, the polymorphisms of *SAA*1/2 gene were associated with cIMT in a large cohort of healthy subjects. This relationship was independent of serum levels of HDL and other determinants of CVD risk. Our observations require further research, but support the notion that the SAA gene directly influences carotid IMT.

## Methods

### Ethical approval of the study protocol

This study was approved by the Ethics Committee of the First Affiliated Hospital of Xinjiang Medical University (Xinjiang, China). It was conducted according to the standards of the Declaration of Helsinki. Written informed consent was obtained from all participants.

### Subjects

The CRS is a prospective, observational cohort study designed to investigate the prevalence, incidence, and risk factors for CVDs and to determine the genetic and environmental contributions to atherosclerosis, CAD and cerebral infarction in the Han, Uygur, and Kazakh population in the Xinjiang province of west China. The CRS involves 14 618 Chinese people (5 757 Hans, 4 767 Uygurs, and 4 094 Kazakhs) aged ≥35 years recruited from seven cities in Xinjiang province: Urumqi, Kelamayi, Hetian, Zhaosu, Fukang, Tulufan, and Fuhai.

Collection of baseline data began in June 2007 and was completed in March 2010. Of 14 618 subjects, 2 318 Han participants were initially screened for the present study and excluded (n = 205) if: systolic and diastolic blood pressure (BP) was ≥140/90 mmHg; fasting plasma glucose ≥7.0 mmol/L; total cholesterol ≥7.8 mmol/L; triglycerides ≥2.0 mmol/L or had electrocardiography (ECG) abnormalities and carotid artery plaques. The final study number was 2113, of which 1914 consented to providing blood samples for DNA analysis. The analysis presented in the present study was based on 1914 subjects (849 men; 1065 women) who had passed the eligibility criteria and had complete data on *SAA*1/2 genotypes.

### Biological and lifestyle measurements

Height and body weight were measured as described previously [26], Sitting blood pressure was measured thrice over 10 min and the median value used in the statistical analysis. The status of smoking and drinking was self-reported using a questionnaire as described previously [27], [28]. Two tubes (one containing ethylenediamine tetra-acetic acid (EDTA) and the other not containing an anticoagulant agent) were used for collecting blood samples after the patient had fasted. The collected samples were separated into plasma, serum and blood cells (including leukocytes) and stored at −80°C for the analysis of routine blood chemistry and DNA extraction. Samples were transported on dry ice at prearranged intervals to laboratories. We measured the serum concentration of total cholesterol, triglyceride, blood urea nitrogen (BUN), creatinine (Cr), LDL, HDL, uric acid and fasting glucose using equipment for chemical analysis (Dimension AR/AVL Clinical Chemistry System, Newark, NJ, USA) employed by the Clinical Laboratory Department of the First Affiliated Hospital of Xinjiang Medical University.

### Carotid ultrasonography

Ultrasonographic evaluation using a 7.5 MHz linear type B-mode probe (Siemens, Berlin, Germany) was undertaken by a specialist to evaluate sclerotic lesions of the common carotid arteries on a day close to the day of blood biochemistry analysis (within 2 days). Details of the procedure are described elsewhere [29].

### Reproducibility study

A reproducibility study was conducted. Two hundred and ten subjects underwent two ultrasound examinations by two sonographers during the same visit; the sonographers were blinded to the study protocol. The mean absolute difference and correlation coefficient between repeated examinations of IMT of the common carotid artery were 0.07mm and 0.84, respectively. For carotid plaques, the Kappa coefficient for agreement between the two examinations was 0.86.

### Genotyping of SAA1/2 SNPs

There are 115 and 76 SNPs for the human SAA1 and SAA2 genes, respectively, listed in the National Center for Biotechnology Information SNP database (http://www.ncbi.nlm.nih.gov/SNP). It has been observed that adjacent SNPs are often highly correlated. To reduce genotyping cost, many algorithms have been developed to select a smallest set of SNPs such that all the other SNPs can be inferred from them. The selected SNPs are called tag SNPs [30]. We selected the tag SNPs of SAA1 and SAA2 gene on the International HapMap Project website using phase I & II database (http://www.hapmap.org/). We obtained two tag SNPs (rs12218 and rs2468844 for the *SAA1* and *SAA2* genes) spanning the coding region of ∼4 kb in *SAA*1 and ∼3 kb in *SAA*2 for Chinese Han subjects using minor allele frequency (MAF) ≥0.05 and linkage disequilibrium patterns with *r* ^2^≥0.8 as a cutoff. Because rs12218 is a synonymous mutation, we selected two additional non-synonymous mutations (rs2229338, rs1059559) across exon 4 of the *SAA1* gene for our analysis. Therefore, in the present study, four SNPs of the *SAA1* and *SAA2* gene were selected for genotype detection. Genomic DNA was extracted from peripheral blood leukocytes using a DNA extraction Kit (Beijing Bioteke Company Limited, Beijing, China). Genotyping was confirmed by polymerase chain reaction (PCR)-restriction fragment length polymorphism (RFLP) analysis. The primers of these four SNPs were designed by use of Primer Premier 5.0. Their synthesis was undertaken by Shanghai Genery Biological Technology Company Limited (Shanghai, China). The primer pair sequences, annealing temperatures, and restriction enzymes for the four SNPs are detailed in Table 6. Digestion of restriction enzymes was according to manufacturer's instructions. To ensure the results to be verified, we used sequenced genomic DNAs as positive controls in our assays.

**Table 6:** Primer sequences of each SNP.

SNPs	Polymerase Chain Reaction Primers	Denaturation temperature	Products length	Restriction enzyme
rs2229338*	Sense: 5′AACAGGGAGAATGGGAGGGTGGG3′	58°C	193bp	*Nco* I
	Antisense: 5′GCAGGTCGGAAGTGATTGGGGTC3′			
rs12218*	Sense: 5′AACAGGGAGAATGGGAGGGTGGG3′	58°C	193bp	*Bgl* I
	Antisense: 5′GCAGGTCGGAAGTGATTGGGGTC3′			
rs1059559*	Sense:5′AACAGGGAGAATGGGAGGGTGGG3′	58°C	193bp	*Tfi* I
	Antisense:5′GCAGGTCGGAAGTGATTGGGGTC3′			
rs2468844	Sense: 5′GGGTCTGAGTGGATGGT3′	55°C	362bp	*Nco* I
	Antisense:5′AGGTCGGAAGTGATTGG3′			

Note:

*Because rs2229338, rs12218, and rs1059559 all locates on the exon 4, we used the same primer pairs.

### Quality control

Of the genotyped samples, 10% were duplicated and there was at least one positive and one negative control per 96-well DNA plate in our assays. The accuracy of the genotyping was determined by genotype concordance between duplicate samples. We obtained 100% concordance between the genotyped duplicate samples for each of the SNPs. The genotyping success rate for each SNP was >98%.

### Statistical analyses

All analyses were carried out using SPSS version 17.0 (SPSS Inc., Chicago, IL, USA). The Hardy-Weinberg equilibrium was assessed using chi-square analysis. Mean values of IMT between the right and left common carotid artery were used in all analyses. These values were normally distributed, so the original values were used for analyses. General linear model analysis was undertaken to test for associations between SNP genotypes and IMT and HDL level after adjusting for confounding variables. Single-SNP effects with continuous variables were analyzed using linear regression using three models. These were the additive (common allele homozygotes coded as 1, heterozygotes as 2, and recessive allele homozygotes as 3); dominant (common allele homozygotes coded as 1 and heterozygotes and recessive allele homozygotesas 2); and recessive (common allele homozygotes and heterozygotes coded as 1 and recessive allele homozygotes as 2) models. Normality was assessed by plotting the residuals. To analyze the interaction between *SAA1* and *SAA2* on IMT, we used the two-way ANOVA method. To assess the association of each SNP with HDL level and IMT, we used a Bonferroni correction to control for the number of variants tested; this was 4, so the probability value, 0.0125, was considered to be significant.
